# Supplementary material for: Experimental evidence of climate change extinction risk in Neotropical montane epiphytes
Source: Nat Commun. 2024 Jul 18;15:6045. doi: 10.1038/s41467-024-49181-5 (PMC11258140; doi:10.1038/s41467-024-49181-5)
Supplement: Supplementary file 1 — Supplementary Information [file 41467_2024_49181_MOESM1_ESM.pdf]

Supplementary Materials for:

Experimental evidence of climate change extinction risk in tropical plants.

Authors: Emily C. Hollenbeck, Dov F. Sax

Correspondence to: hollenbeck.ec@gmail.com

This file includes:

Figs. S1 to S11
Tables S1 to S8

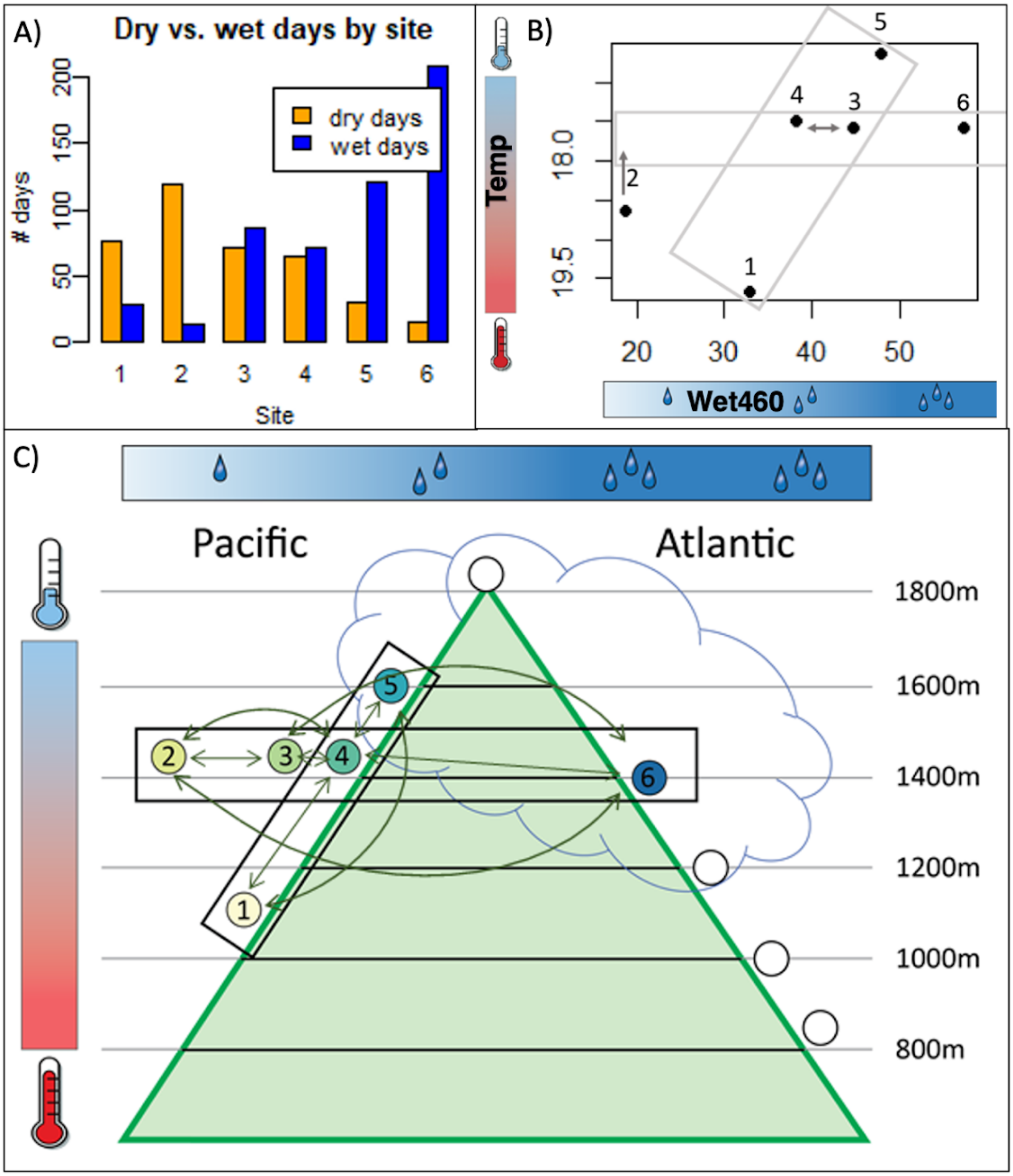

Fig. S1. Climatic gradients in Monteverde provided the context for our transplant experiment and range surveys. Sites 1-6 were used for the transplant experiment. White circles represent additional sites included in range surveys (Fig. S3). A) Total number of dry and wet days during an 11-month period. B) Temperature (y axis; inverse so cooler temperatures are higher) vs. average leaf wetness (LWS) (x axis, "Wet460") during an 11-month period. See methods for details on climate data collection and calculations. C) Study sites illustrated along qualitative representations of the climate gradients quantified in A) and B), as well as elevational gradients. Each box (in B and C) shows a transect along which transplants took place: a

horizontal “moisture” transect, with four sites (2, 3, 4, and 6) around the same elevation, so they have similar temperature profiles (although site 2 appears to be warmer, B), but vary greatly in their moisture availability. The Pacific elevational transect (sites 1, 4, and 5) crosses gradients of both temperature and moisture availability, but the change in moisture is not as dramatic as that along the horizontal transect. Transplants were moved reciprocally between all sites along each gradient. Each arrow represents a “transplant direction,” where transplants moved between a particular site pair.

We note that sites along our aridity transect (sites 2, 3, 4 and 6) exhibit a pronounced gradient in leaf wetness, but that the relative position of sites 3 and 4 along this gradient is dependent on the specific measure of aridity examined. Based on an index that incorporates temperature and average leaf wetness, site 4 is more arid than site 3 (Panel B). This relative position is swapped when considering the total number of dry days (Panel A). This latter measure is more consistent with our qualitative perception of dryness. This measure is also consistent with species occurrence data, with many mesic species (found in cloud forest) present at site 4 but not site 3, and with survival data, as several species had survival boundaries such that they survived at site 4 and wetter, but not at site 3 and drier. Of course, different species might be sensitive to different aspects of aridity and no single measure is best, particularly when the relative difference between two sites is minimal. We performed our primary analysis of survival versus aridity (Figs. 2, S4) based on the values shown in Panel B, but found that our overall results are robust to the position of these two sites.

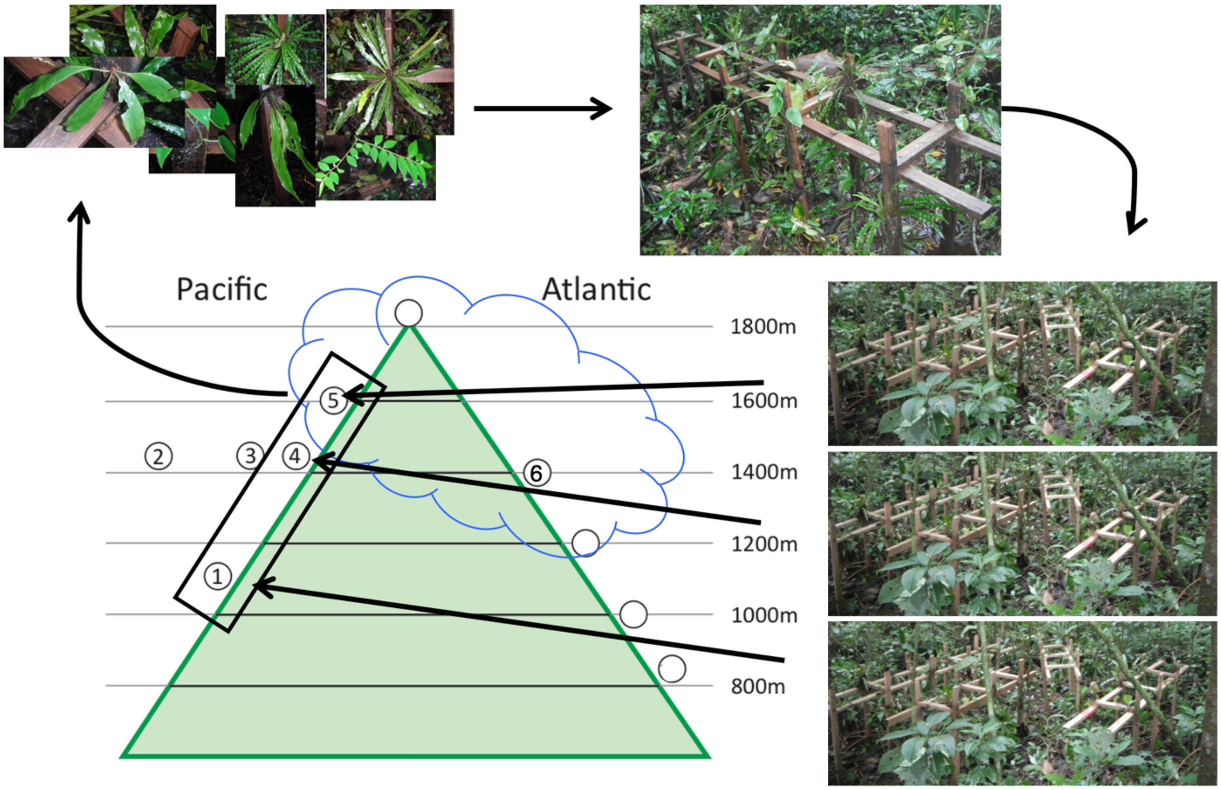

Fig. S2. Illustration of transplant experimental process. Using site 5 as an example, the seven species appropriate for the transplant experiment at this site (see Methods) were collected in the forest understory and attached to wooden scaffolds (actual examples pictured, photos taken by the authors). Four identical replicate scaffolds were created for each destination site along the gradient; in this case, to sites 1, 4, and 5. The scaffolds returned to their native site 5 are the experimental controls.

a) Monteverde, C.R.

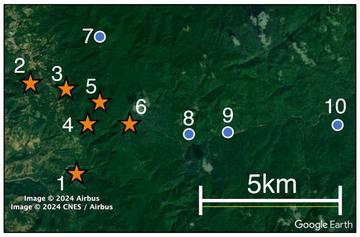

Site no.	Elevation (m.a.s.l.)	Slope
1	1100	Pacific
2	1380	Pacific
3	1400	Pacific
4	1480	Pacific
5	1600	Pacific
6	1400	Atlantic
7	1800	Cont. div.
8	1200	Atlantic
9	1000	Atlantic
10	850	Atlantic

b) Barva, C.R.

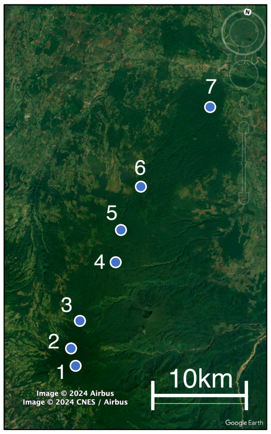

Site no.	Elevation (m.a.s.l.)
1	2800
2	2500
3	2000
4	1500
5	1000
6	500
7	30

c) Baru, Panama

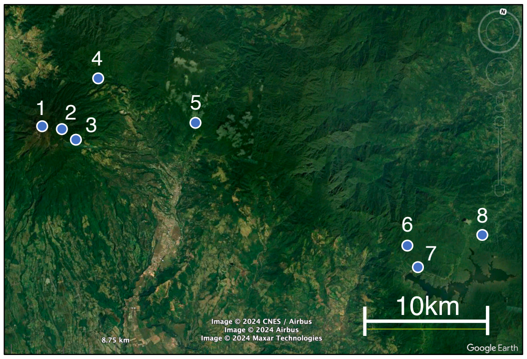

Site no.	Elevation (m.a.s.l.)
1	3400
2	3000
3	2600
4	2000
5	1700
6	1500
7	1200
8	800

Fig. S3. **Maps of surveyed elevational gradients.** Colored points show the exact locations of survey sites on three mountains in Costa Rica and western Panama, with their elevations listed below. Transplant sites in Monteverde are illustrated with orange stars. Map images are sourced from Google Earth, site locations were embedded in Google Earth from GPS points using a Garmin GPS device, and images were edited to improve clarity of site numbers and point size using Adobe Illustrator.

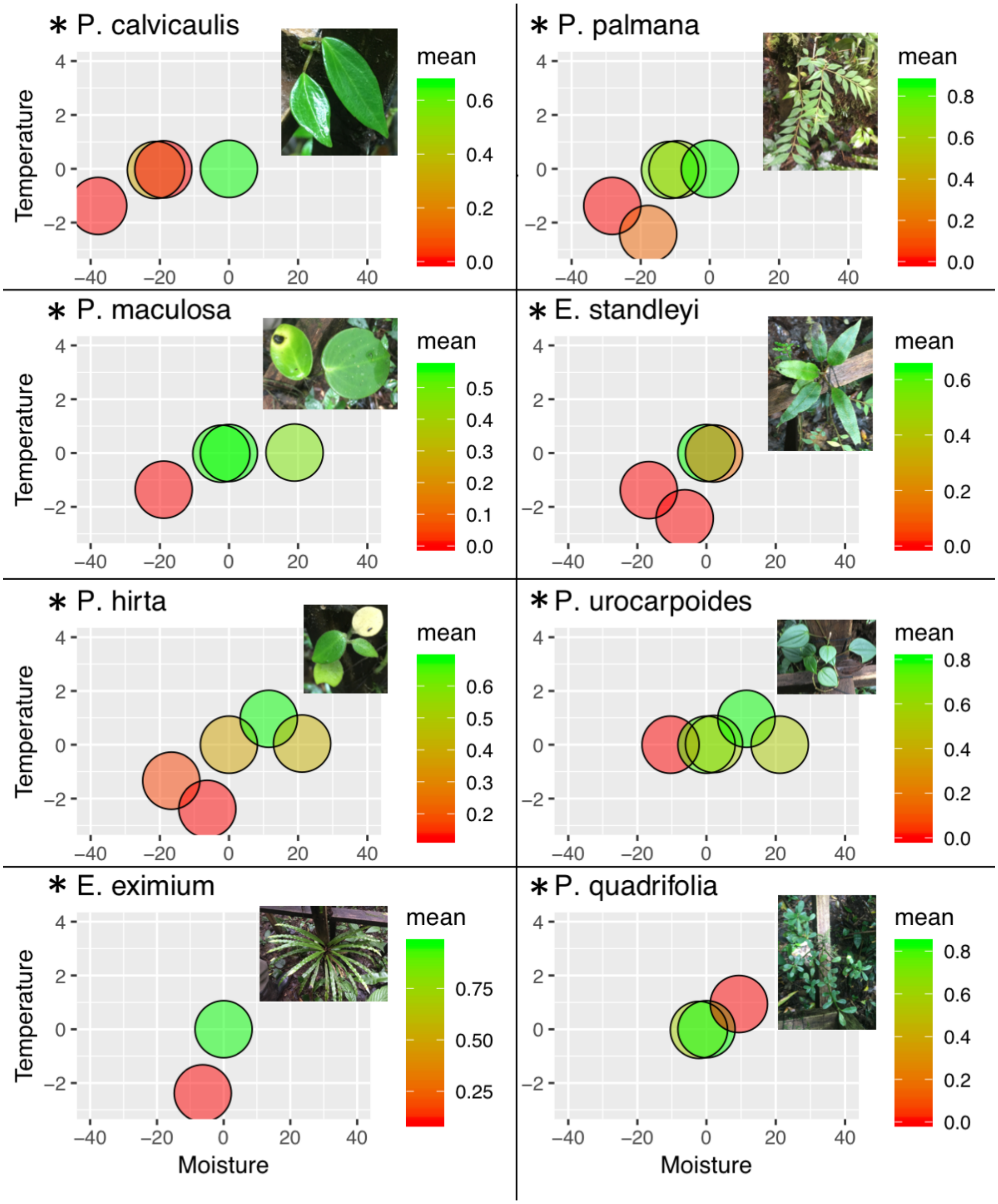

Fig. S4. Individual species' results from the 3-year transplant experiment. Temperature and moisture axes are plotted as in Figure 2; axes are identical for all species. Circles represent a set of replicates of that species transplanted at a climatic distance from the native range boundary as indicated by position along the x (moisture; LWS) and y (Temperature) axes; all transplants within the native range are at (0,0). Circle color indicates mean percent survival at that site; note that species have different color scales. Asterisks indicate species for which Logrank tests of K-M survival curves indicated a significant effect of destination site on transplant survival with $p < 0.05$ (Table S2; see Fig S6 for an example). Source data are provided as a Source Data file. All photos taken by the authors.

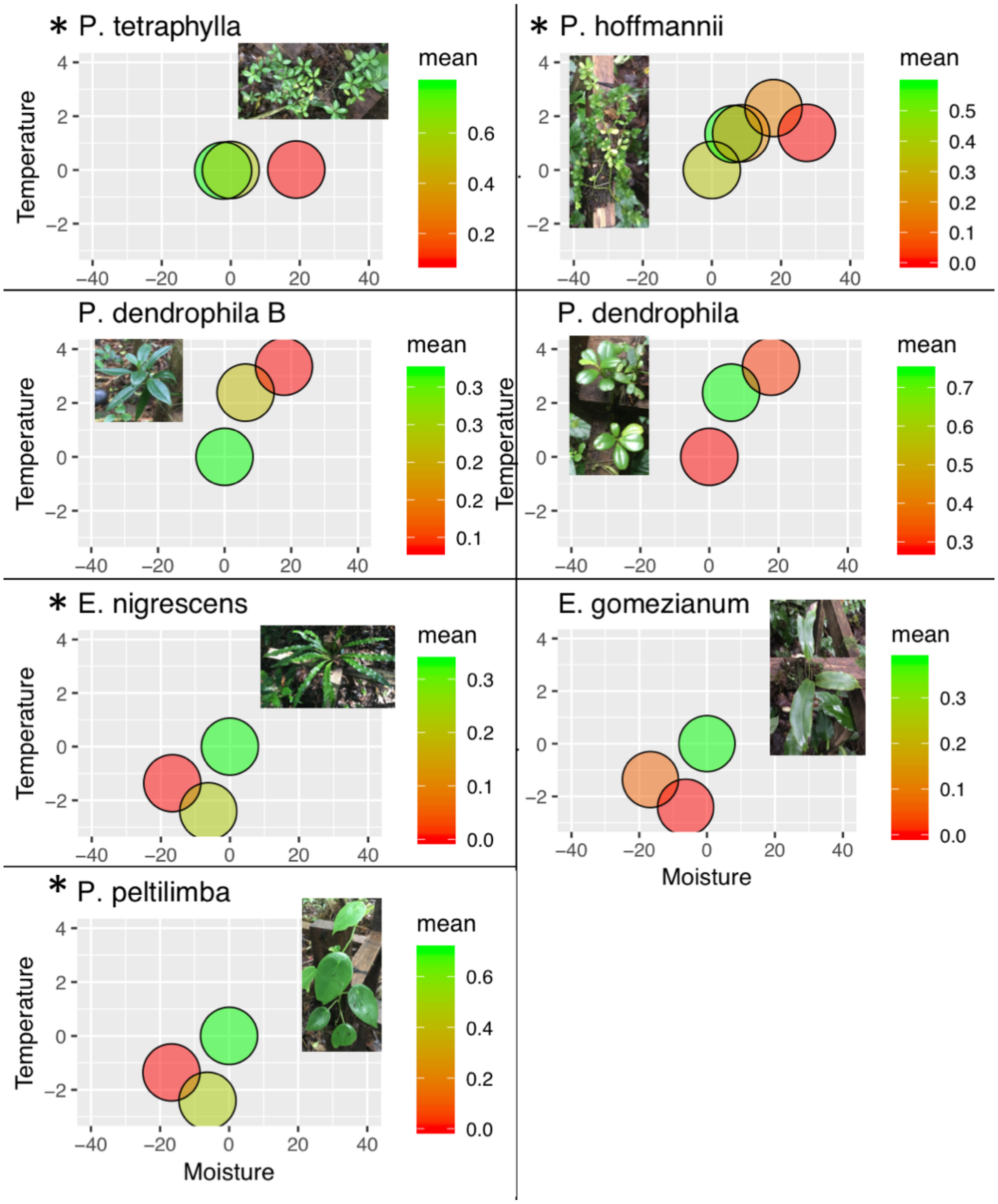

Fig. S4 continued. Individual species' results from the 3-year transplant experiment (continued). Temperature and moisture axes are plotted as in Figure 2; axes are identical for all species. Circles represent a set of replicates of that species transplanted at a climatic distance from the native range boundary as indicated by position along the x (moisture; LWS) and y (Temperature) axes; all transplants within the native range are at (0,0). Circle color indicates mean percent survival at that site; note that species have different color scales. Asterisks indicate species for which Logrank tests of K-M survival curves indicated a significant effect of destination site on transplant survival with $p < 0.05$ (Table S2; see Fig S6 for an example). Source data are provided as a Source Data file.

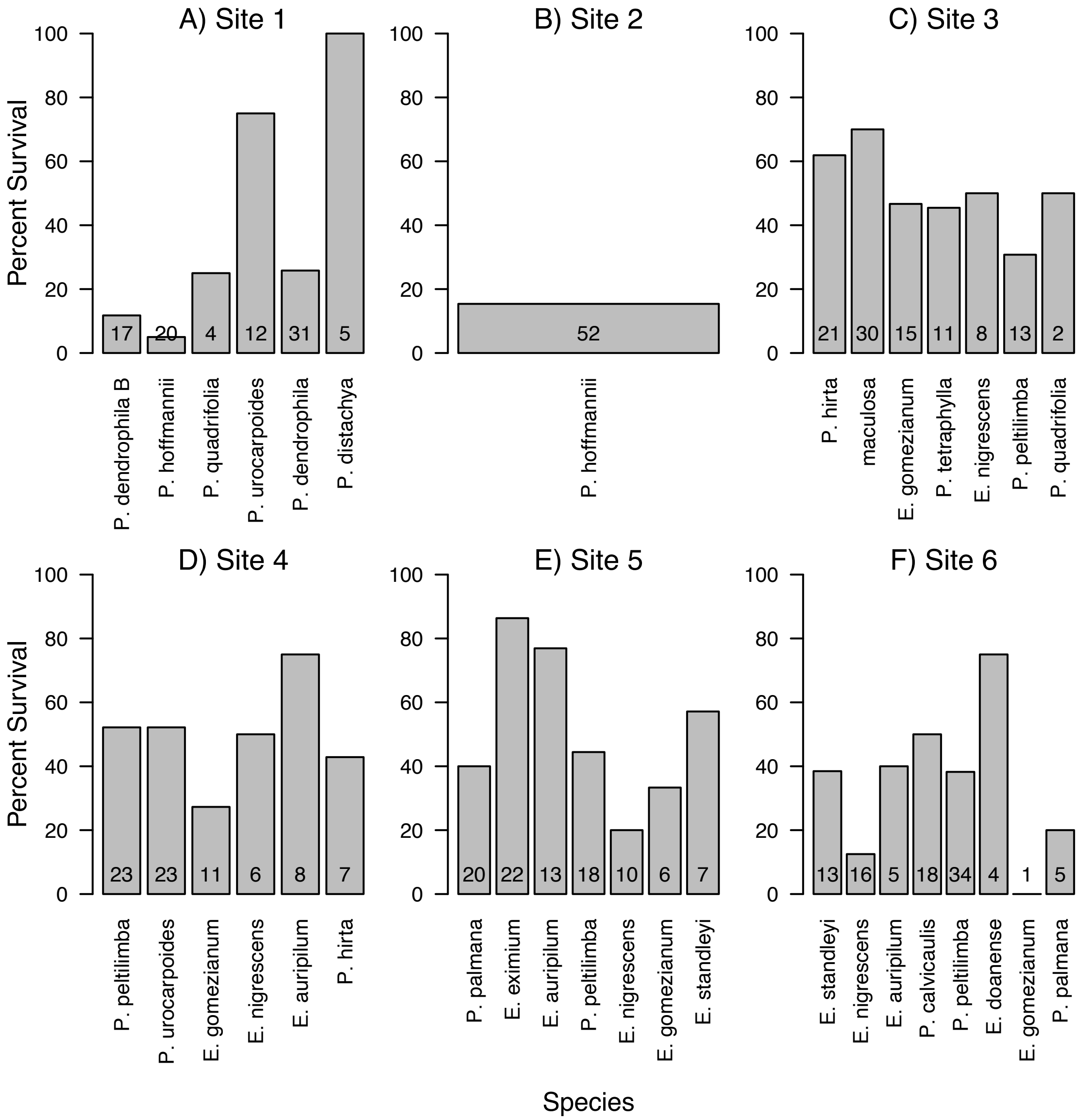

Fig. S5. Survival of unmanipulated forest control plants was low, species-dependent and highly idiosyncratic. Percent survival (y-axis) is shown for each species (x-axis bars) monitored as forest controls at each site (panels A-F represent sites 1-6, respectively). Numbers on bars indicate sample size, e.g. initial number of control plants alive per species at each site at the beginning of the experiment. Error bars are not included because there is no replication; survival values are simply the % of individuals alive at the end of the experiment.

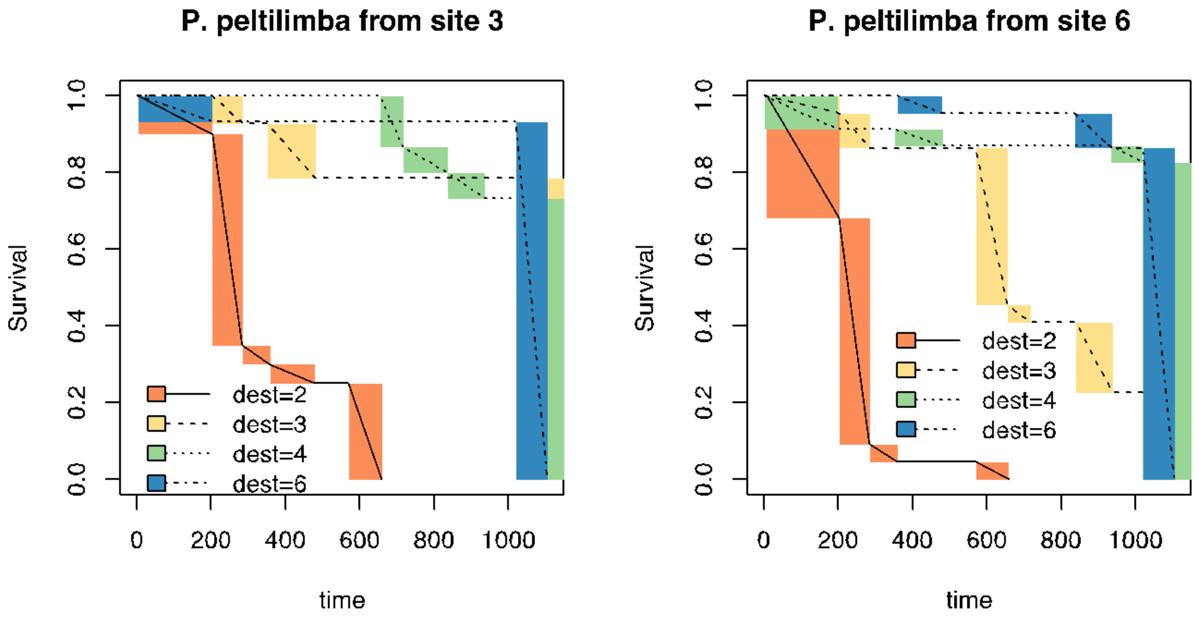

Fig. S6. ***Peperomia peltilimba*** **population of origin has an impact on transplant survival** at a destination site (3) on the dry periphery of the native range. Kaplan-Meier curves are illustrated over time (x axis shows days after transplant) for each transplant destination (colors) coming from origin sites 3 and 6 (two panels). Transplants from site 3 that went to destination sites 3, 4, and 6 are not statistically different from each other. However, transplants from site 6 suffered significantly lower survival at site 3 than sites 4 and 6 (Table S3). This indicates that the population from site 6, which is wetter, was unable to survive as well in the drier site 3 – even though conspecific populations native to site 3 had equally high survival throughout the range. In the left panel, for *P. peltilimba* originating at site 3, sample size in number of individuals for each transplant direction (destination site, or unique lines) was 19, 14, 15, and 16 for destinations 2, 3, 4 and 6, respectively. In the right panel, for *P. peltilimba* originating at site 6, sample size in number of individuals for each transplant direction was 22, 21, 22, and 21, for destinations 2, 3, 4 and 6, respectively.

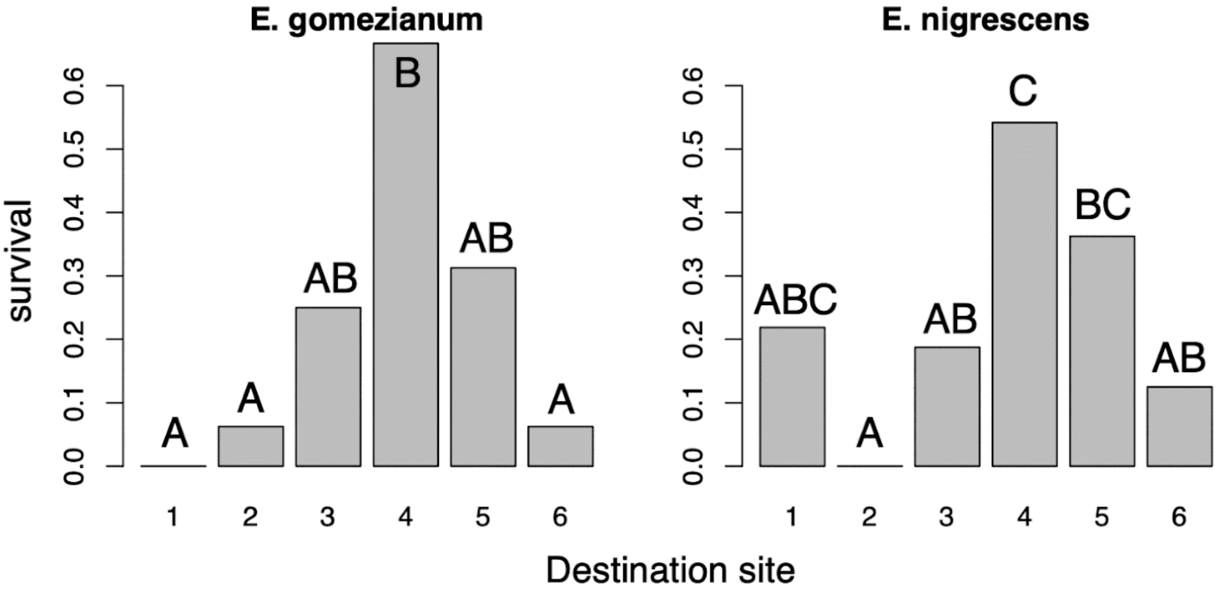

Fig. S7. ***Elaphoglossum gomezianum* and *E. nigrescens* have reduced survival at both wet and dry peripheries of their native range**, compared to the most central part of their range along the studied transects. Bars illustrate the fraction of plants alive (y axis) of all individuals from each species after 3 years post-transplant to each destination site. Both species were found growing naturally at all sites except site 2. For *E. gomezianum*, survival was significantly higher at site 4 than at sites 1, 2, and 6, while for *E. nigrescens*, site 4 survival was significantly higher than sites 2, 3, and 6, while survival at site 5 was significantly higher than at site 2. Significance of destination site was determined by a one-way ANOVA, and pairwise significant differences between sites were calculated with a Tukey HSD (Table S4). For *E. gomezianum*, sample sizes in number of individuals at each destination site were 8, 13, 13, 16, 13, and 13, for sites 1-6 in order. For *E. nigrescens*, sample sizes in number of individuals at each destination site were 26, 38, 40, 56, 32, and 40, for sites 1-6 in order.

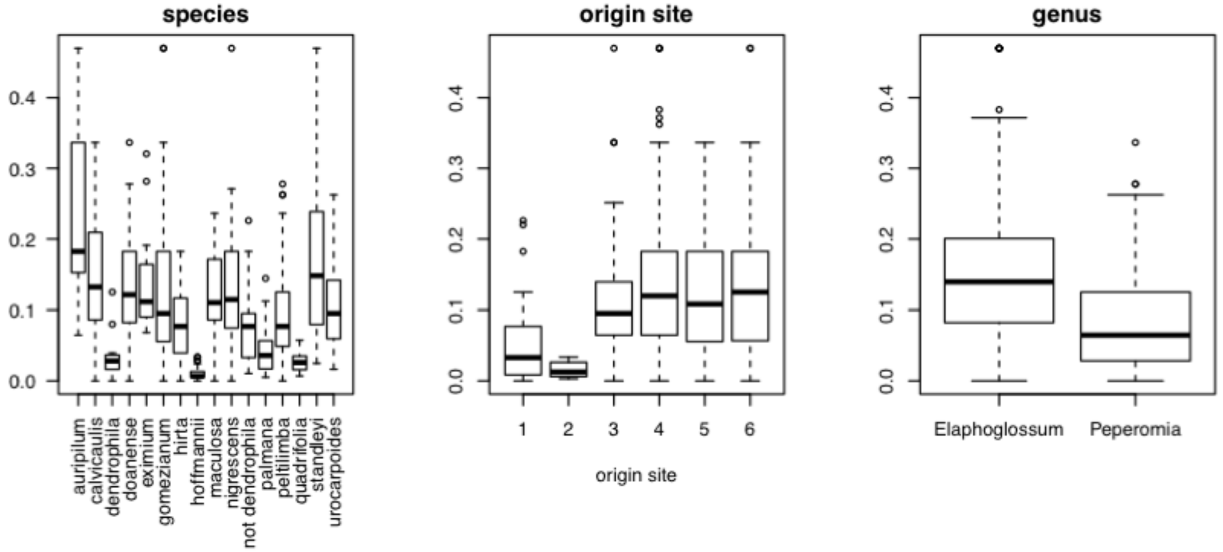

Fig. S8. Baseline herbivory by species, origin site, and genus. All three factors explain significant variation in baseline herbivory according to ANOVA (see Table S5). Each box plot illustrates the herbivory levels at the beginning of the experiment for all plants within each category; the Y axis is log (fraction herbivory + 1). Raw data were transformed with log(+1) prior to visualization and statistical tests.

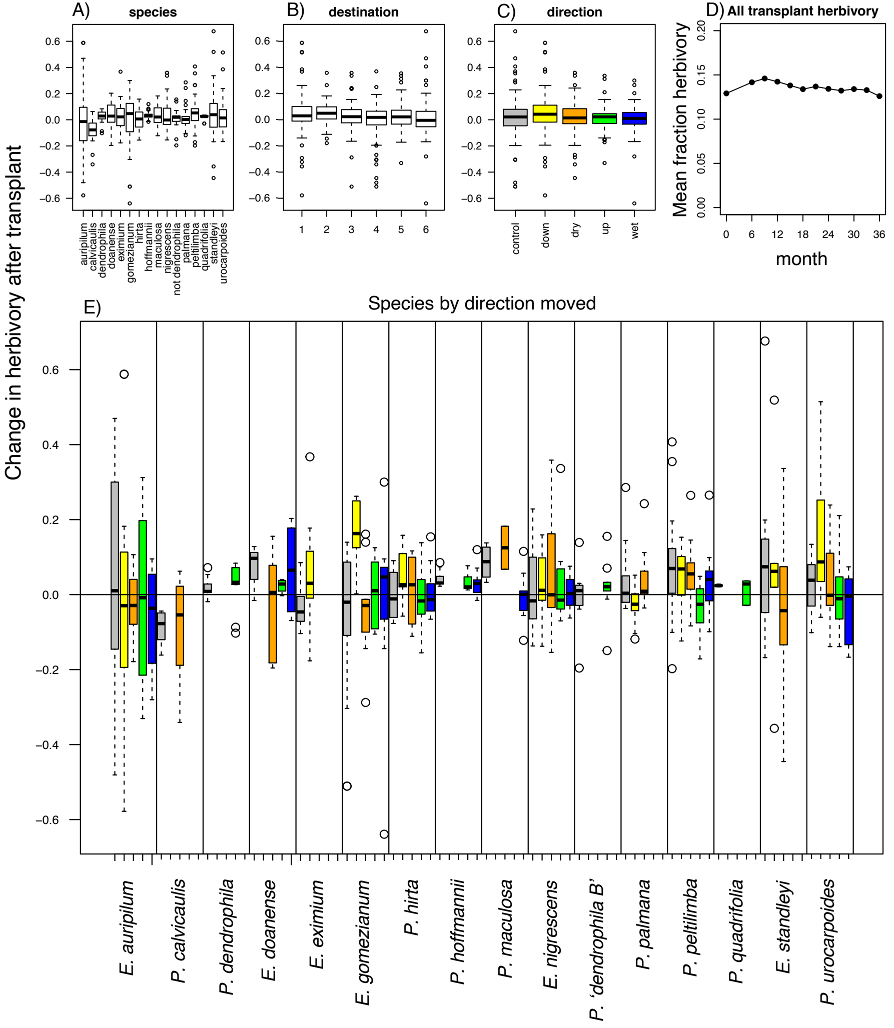

Change in herbivory after transplant
A) species
auripilum
calvicaulis
dendrophila
doanense
eximium
gomezianum
hirta
hoffmannii
maculosa
nigrescens
not dendrophila
palmana
peltilimba
quadrifolia
standleyi
urocarpoides
B) destination
1
2
3
4
5
6
C) direction
control
down
dry
up
wet
D) All transplant herbivory
Mean fraction herbivory
month
E) Species by direction moved
E. auripilum
P. calvicaulis
P. dendrophila
E. doanense
E. eximium
E. gomezianum
P. hirta
P. hoffmannii
P. maculosa
E. nigrescens
P. 'dendrophila B'
P. palmana
P. peltilimba
P. quadrifolia
E. standleyi
P. urocarpoides

Fig. S9. Change in herbivory after 3 years post-transplant. There was no significant effect of species, destination, direction, or species * direction on the change in herbivory from the beginning to the end of the experiment (Table S5). In panels A-C and E, each box plot illustrates the change in herbivory for all plants within each category that survived to the end of the experiment. Colors in panel E indicate transplant direction as labeled in C. The Y axis is log ((difference in fraction herbivory from first to last timepoint) + 1). Raw data were transformed with log(+1) prior to visualization and statistical tests. Panel D shows mean herbivory (raw fraction herbivory values) in all transplants over time during the experiment.

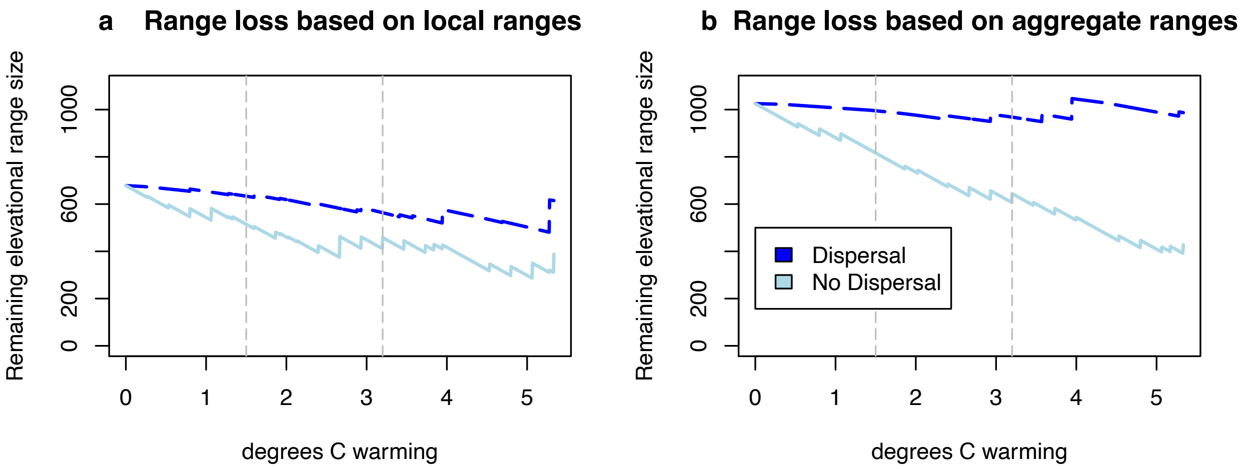

Fig. S10. Elevational range size of populations (a) and species (b) decreases as warming increases. Alternate scenarios consider full upward dispersal (dark blue) vs. no dispersal (light blue). Elevational ranges are calculated from local populations (a) vs. species aggregate (b) ranges (methods). Lines show the remaining elevational range of extant species, highlighting 1.5°C and 3.2°C (dashed vertical lines). Sharp increases occur as species with declining range sizes become extirpated as their range size declines to 0, removing them from the pool of range size calculated.

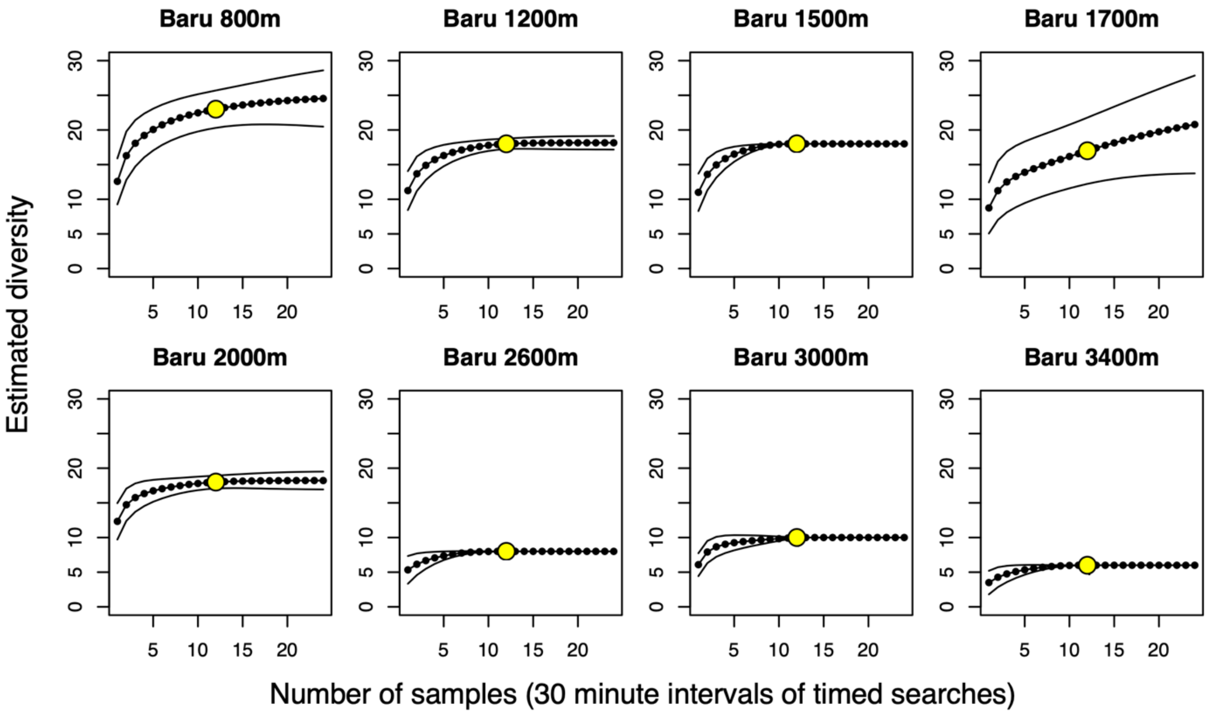

Fig. S11. Species-accumulation curves (SACs) for Baru sites calculated using EstimateS software. Points indicate the estimated diversity after a given number of sampling units; yellow dot is actual sampling effort, and lower sample numbers are interpolated while higher sample numbers are extrapolated. Asymptote is estimated actual diversity. Solid lines indicate 95% confidence interval.

Table S1.
Sample sizes for transplant experiment. N indicates the number of individuals moved of each species from each unique site of origin to site of destination. “Origin” and “destination” refer to site numbers from Monteverde used throughout the study.

genus	species	origin	destination	N
E.	*eximium*	5	1	20
E.	*eximium*	5	4	20
E.	*eximium*	5	5	20
E.	*gomezianum*	3	2	9
E.	*gomezianum*	3	3	9
E.	*gomezianum*	3	4	8
E.	*gomezianum*	3	6	9
E.	*gomezianum*	4	1	4
E.	*gomezianum*	4	2	4
E.	*gomezianum*	4	3	4
E.	*gomezianum*	4	4	4
E.	*gomezianum*	4	5	7
E.	*gomezianum*	4	6	4
E.	*gomezianum*	5	1	4
E.	*gomezianum*	5	4	4
E.	*gomezianum*	5	5	6
E.	*nigrescens*	3	2	7
E.	*nigrescens*	3	3	9
E.	*nigrescens*	3	4	7
E.	*nigrescens*	3	6	8
E.	*nigrescens*	4	1	15
E.	*nigrescens*	4	2	16
E.	*nigrescens*	4	3	16
E.	*nigrescens*	4	4	16
E.	*nigrescens*	4	5	19
E.	*nigrescens*	4	6	16
E.	*nigrescens*	5	1	10
E.	*nigrescens*	5	4	17
E.	*nigrescens*	5	5	12
E.	*nigrescens*	6	2	14
E.	*nigrescens*	6	3	14
E.	*nigrescens*	6	4	15
E.	*nigrescens*	6	6	15

E.	*standleyi*	5	1	4
E.	*standleyi*	5	4	4
E.	*standleyi*	5	5	4
E.	*standleyi*	6	2	6
E.	*standleyi*	6	3	7
E.	*standleyi*	6	4	7
E.	*standleyi*	6	6	7
P.	*calvicaulis*	6	2	20
P.	*calvicaulis*	6	3	20
P.	*calvicaulis*	6	4	24
P.	*calvicaulis*	6	6	18
P.	*dendrophila*	1	1	18
P.	*dendrophila*	1	4	16
P.	*dendrophila*	1	5	16
P.	*hirta*	3	2	13
P.	*hirta*	3	3	12
P.	*hirta*	3	4	13
P.	*hirta*	3	6	12
P.	*hirta*	4	1	8
P.	*hirta*	4	2	9
P.	*hirta*	4	3	8
P.	*hirta*	4	4	9
P.	*hirta*	4	5	11
P.	*hirta*	4	6	10
P.	*hoffmannii*	1	1	16
P.	*hoffmannii*	1	4	11
P.	*hoffmannii*	1	5	14
P.	*hoffmannii*	2	2	20
P.	*hoffmannii*	2	3	21
P.	*hoffmannii*	2	4	19
P.	*hoffmannii*	2	6	21
P.	*maculosa*	3	2	13
P.	*maculosa*	3	3	14
P.	*maculosa*	3	4	15
P.	*maculosa*	3	6	14
P.	*"dendrophila B"*	1	1	16
P.	*"dendrophila B"*	1	4	21
P.	*"dendrophila B"*	1	5	21
P.	*palmana*	5	1	16

P.	*palmana*	5	4	15
P.	*palmana*	5	5	16
P.	*palmana*	6	2	8
P.	*palmana*	6	3	8
P.	*palmana*	6	4	9
P.	*palmana*	6	6	6
P.	*peltilimba*	3	2	20
P.	*peltilimba*	3	3	14
P.	*peltilimba*	3	4	15
P.	*peltilimba*	3	6	16
P.	*peltilimba*	4	1	20
P.	*peltilimba*	4	2	22
P.	*peltilimba*	4	3	20
P.	*peltilimba*	4	4	19
P.	*peltilimba*	4	5	20
P.	*peltilimba*	4	6	20
P.	*peltilimba*	5	1	22
P.	*peltilimba*	5	4	20
P.	*peltilimba*	5	5	21
P.	*peltilimba*	6	2	22
P.	*peltilimba*	6	3	21
P.	*peltilimba*	6	4	22
P.	*peltilimba*	6	6	21
P.	*quadrifolia*	1	1	4
P.	*quadrifolia*	1	4	6
P.	*quadrifolia*	1	5	6
P.	*tetraphylla*	3	2	12
P.	*tetraphylla*	3	3	12
P.	*tetraphylla*	3	4	13
P.	*tetraphylla*	3	6	13
P.	*urocarpoides*	1	1	14
P.	*urocarpoides*	1	4	12
P.	*urocarpoides*	1	5	13
P.	*urocarpoides*	4	1	20
P.	*urocarpoides*	4	2	19
P.	*urocarpoides*	4	3	20
P.	*urocarpoides*	4	4	21
P.	*urocarpoides*	4	5	20
P.	*urocarpoides*	4	6	21

Table S2.
Logrank test of the impact of destination site on each species' KM survival curve. Species for which transplant destination had a significant impact on survival with $p<0.05$ are shown in bold. Marginally significant (*E. nigrescens*, p=0.0847) shown with underline.

**genus**	**species**	**p value**	**Z statistic**
P.	***calvicaulis***	**9.21E-13**	-7.141775258
E.	***nigrescens***	**2.39E-02**	-2.258707246
P.	***peltilimba***	**5.96E-22**	-9.630167215
P.	***palmana***	**9.17E-06**	-4.435922403
E.	***standleyi***	**3.00E-04**	-3.615024448
E.	***eximium***	**1.14E-10**	-6.447332908
E.	*gomezianum*	*8.47E-02*	-1.723985869
P.	***urocarpoides***	**1.49E-02**	-2.433640519
P.	***hirta***	**1.01E-02**	-2.573918508
P.	***maculosa***	**1.40E-02**	-2.456044919
P.	***tetraphylla***	**1.05E-02**	2.560280639
P.	***hoffmannii***	**5.02E-03**	2.805637173
P.	*dendrophila*	2.17E-01	-1.233548622
P.	*"dendrophila B"*	2.03E-01	1.271835308
P.	***quadrifolia***	**1.00E-02**	NA

Table S3.
Pairwise comparisons of Asymptotic Logrank two-sample tests for *P. peltilimba* transplants to destination sites from different sites of origin. These correspond to the data illustrated in Fig S8. Bold rows indicate significant pairwise differences. The first table shows that for *P. peltilimba* individuals sourced from site 3 and transplanted to sites 2, 3, 4, and 6, survival was significantly lower at site 2 than at all other sites, but there were no significant differences between survival at destination sites 3, 4, and 6. The bottom table shows that, in contrast, for individuals sourced from site 6 (the wettest site), in addition to reduced survival at site 2, their survival was also significantly lower at site 3 compared to site 6. Survival at site 4 was not significantly different from either site 3 or site 6.

Site of origin: 3		
pairs	score statistic	p
**2 and 3**	9.14	**6.10E-06**
**2 and 4**	10.5	**5.80E-07**
**2 and 6**	10.15	**3.00E-07**
3 and 6	1.05	2.80E-01
3 and 4	0.19	8.80E-01
4 and 6	1.52	1.70E-01

Site of origin: 6		
pairs	score statistic	p
**2 and 3**	11.2	**7.90E-06**
**2 and 4**	11.96	**6.80E-08**
**2 and 6**	13.02	**3.30E-09**
**3 and 6**	8.66	**2.60E-05**
3 and 4	7.89	2.50E-04
4 and 6	0.54	6.80E-01

Table S4: ANOVA and Tukey HSD results showing impact of destination site on survival of transplants. We found a statistically significant effect of destination site (p=0.001), but not origin site or the interaction of origin * destination, in the survival of both *E. gomezianum* and *E. nigrescens* after 3 years post-transplant, using a two-way ANOVA. Arcsin transformation was applied to percentage survival (dependent variable) prior to analysis. Pairwise differences are shown, calculated with Tukey HSD post-hoc test from a one-way ANOVA measuring the impact of destination site on 3-year survival of each species. Significant differences are shown in bold. Data visualized in Figure S9.

E. gomezianum

One-way ANOVA

	Df	Sum of squares	Mean square	F value	p value
**destination**	5	7.827	1.5655	5.237	**0.0007**
Residuals	46	13.751	0.2989		

Two-way ANOVA

	Df	Sum of squares	Mean square	F value	p value
origin	2	0.219	0.1097	0.36	0.6997
**destination**	5	7.617	1.5234	5.003	**0.0012**
origin * destination	5	1.867	0.3734	1.226	0.3152
Residuals	39	11.874	0.3045		

Tukey HSD pairwise comparisons

site pairs	mean difference	lower bound	upper bound	p value
2-1	0.098	-0.715	0.911	0.9992
3-1	0.393	-0.420	1.205	0.7050
**4-1**	**1.047**	**0.305**	**1.789**	**0.0016**
5-1	0.491	-0.322	1.304	0.4785
6-1	0.098	-0.715	0.911	0.9992
3-2	0.295	-0.518	1.107	0.8878
**4-2**	**0.949**	**0.207**	**1.691**	**0.0053**
5-2	0.393	-0.420	1.205	0.7050
6-2	0.000	-0.813	0.813	1.0000
4-3	0.654	-0.087	1.396	0.1122
5-3	0.098	-0.715	0.911	0.9992

E. nigrescens

One-way ANOVA

	Df	Sum of squares	Mean square	F value	p value
**destination**	5	5.316	1.0631	5.731	**0.0002**
Residuals	62	11.5	0.1855		

Two-way ANOVA

	Df	Sum of squares	Mean square	F value	p value
origin	3	1.154	0.3848	1.98	0.1286
**destination**	5	4.534	0.9069	4.667	**0.0014**
origin * destination	8	1.217	0.1521	0.783	0.6197
Residuals	51	9.91	0.1943		

Tukey HSD pairwise comparisons

site pairs	mean difference	lower bound	upper bound	p value
2-1	-0.360	-0.938	0.218	0.4538
3-1	-0.055	-0.633	0.524	0.9998
4-1	0.452	-0.096	1.001	0.1634
5-1	0.184	-0.450	0.817	0.9560
6-1	-0.142	-0.720	0.436	0.9786
3-2	0.305	-0.212	0.822	0.5132
**4-2**	**0.812**	**0.329**	**1.296**	**0.0001**
**5-2**	**0.544**	**-0.034**	**1.122**	**0.0769**
6-2	0.218	-0.299	0.735	0.8150
**4-3**	**0.507**	**0.023**	**0.991**	**0.0347**
5-3	0.238	-0.340	0.816	0.8294

6-3	-0.295	-1.107	0.518	0.8878	6-3	-0.087	-0.604	0.430	0.9961
5-4	-0.556	-1.298	0.186	0.2445	5-4	-0.269	-0.817	0.280	0.7023
**6-4**	**-0.949**	**-1.691**	**-0.207**	**0.0053**	**6-4**	**-0.594**	**-1.078**	**-0.111**	**0.0077**
6-5	-0.393	-1.205	0.420	0.7050	6-5	-0.326	-0.904	0.253	0.5655

Table S5.

ANOVA results for herbivory. Herbivory rates were transformed with log (+1) prior to analysis and visualization. We show that species, origin site, genus, and the interaction of genus * origin significantly impact baseline, natural herbivory rates in this system (p values in table below; significant values of $p<0.05$ in bold). However, the transplant experiment did not cause any systematic changes in herbivory; no significant effects were detected from species, direction of movement, destination site, or the interactions between species * direction or species * destination on changes in herbivory levels during the experiment (although the effect of destination site was marginally significant with $p=0.055$). Overall herbivory did not change throughout the 3-year course of the experiment. Thus, we conclude that herbivory pressure did not contribute to differential survival of species moved outside their native range.

Baseline herbivory

Impact on baseline herbivory:

	Independent variable	Df	Sum Sq	Mean Sq	F value	p
1-way ANOVAs	**species**	15	1.504	0.10028	16.29	**<2e-16**
	**origin site**	5	0.532	0.10637	13.17	**4.98E-12**
	**genus**	1	0.76	0.7605	101	**<2e-16**
2-way ANOVAs	species * origin	11	0.0852	0.00775	1.269	0.239
	**genus * origin**	3	0.06	0.02	2.817	**0.0387**

Change in herbivory

Impact on change in herbivory:

	Independent variable	Df	Sum Sq	Mean Sq	F value	p
1-way ANOVAs	species	15	0.364	0.02425	1.426	0.129
	destination	5	0.186	0.03713	2.182	0.0547
	direction	4	0.109	0.02735	1.598	0.173
2-way ANOVAs	species * destination	54	1.017	0.01884	1.129	0.253
	species * direction	36	0.767	0.02129	1.285	0.128

Table S6.
Species by site occurrence matrix for Barva. Each row is a species, each column a survey site, indicated by elevation in m.a.s.l. Numbers in each cell represent the average number of individuals of each species found in each 30-minute interval of timed searches.

	**elevation**	**2800**	**2500**	**2000**	**1500**	**1000**	**500**	**30**
E.	*alfredii*	26.5	103.8	0	0	0	0	0
E.	*conspersum*	9.9	5.9	0	0	0	0	0
E.	*furfuraceum*	26.3	1.3	0.1	0	0	0	0
E.	*papillosum*	24.8	0.3	0	0	0	0	0
E.	*squamipes*	4.2	0	0	0	0	0	0
E.	*lehmannianum*	0.7	0	0.1	0	0	0	0
P.	*galioides*	0.3	0	0	0	0	0	0
E.	*moranii*	0.1	0.1	8.8	0.4	1.5	0	0
E.	*hammellianum*	0	1.6	0.3	0	1	0	0
E.	*peltatum*	0	35.2	20.4	11	0.6	8.6	0.4
E.	*subcuspidatum*	0	2.8	8.7	0	0	0	0
E.	*eximium*	0	0.1	89.3	4.5	0	0	0
E.	*gloeorrhizum*	0	2.3	4.8	0	0	0	0
E.	*lingua*	0	0	1.4	0	0	0	0
E.	*auripilum*	0	0	0.6	0	0	0	0
E.	*proximum*	0	0	0.5	5.8	0.25	0	0
P.	*palmana*	0	0	5.6	1.8	0.5	0	0
P.	*pittieri*	0	0	7.4	8.3	0	0	0
P.	*hernandiifolia*	0	0	14.3	18.4	12.4	0	0
P.	*peltilimba*	0	0	0.6	0	0	0	0
E.	*decoratum*	0	0	0	0.9	0.2	0	0
E.	*doanense*	0	0	0	0.1	0	4.1	0
E.	*nigrescens*	0	0	0	0	2.8	0.4	2.1
P.	*serpens*	0	0	0	0	3.8	4.3	1.7
P.	*urocarpoides*	0	0	0	0	0.1	0	0
E.	*mitorrhizum*	0	0	0	0	6.0	0	0
E.	*crinitum*	0	0	0	0	0.1	0.25	0
E.	*phoras*	0	0	0	0	1.0	0	0
E.	*smithii*	0	0	0	0	5.4	0	0
E.	*ciliatum*	0	0	0	0	0.1	0	0
P.	*montium*	0	0	0	0	0.6	0.3	0
E.	*lalitae*	0	0	0	0	0.1	2.9	0
P.	*rotundifolia*	0	0	0	0	0.1	0.1	0
E.	*boragineum*	0	0	0	0	0	35.3	0
E.	*siliquoides*	0	0	0	0	0	4.3	0

E. backhousianum	0	0	0	0	0	0.2	0
E. brenesii	0	0	0	0	0	1.0	0
E. heterochroum	0	0	0	0	0	2.9	0
P. distachya	0	0	0	0	0	0.9	0
E. herminieri	0	0	0	0	0	0.8	2.2
P. flexinerva	0	0	0	0	0	0.2	0.3
E. productum	0	0	0	0	0	0.1	0.3

Table S7.

Species by site occurrence matrix for Monteverde. Slope is designated because Monteverde sites fall along more than one elevational and climatic gradient (Fig. 2, Fig S1). Site numbers refer to the transplant experiment. Each row is a species, each column a survey site, indicated by elevation in m.a.s.l. Numbers in each cell represent the average number of individuals of each species found each 30-minute interval of timed searches.

slope		Pacific					Divide	Atlantic			
site number		1	2	3	4	5		6			
elevation		1100	1380	1450	1480	1600	1800	1400	1200	1000	850
P.	dendrophila	92	0	0	0	0	0	0	0	0	0
P.	"dendrophila B"	185	0	0	0	0	0	0	0	0	0
P.	hoffmannii	139	88	1	0	0	0	0	0	0	0
P.	tetraphylla	2	20	65	0	0	0	0	0	0	0
E.	glabellum	10	0	31	0	0	0	0	0	0	0
P.	quadrifolia	57	9	43	4	0	0	0	0	0	0
P.	rotundifolia	45	0	9	5	0	0	0	0	249	43
E.	hammelianum	22	0	30	85	17	0	91	36	57	12
E.	caricifolium	36	0	28	0.5	0	0	0	0.5	25	32
P.	urocarpoides	58	0	0	204	0	0	0	0	169	59
P.	distachyos	0.5	0	0	4	7	0	135	82	162	154
E.	doanense	0.5	0	0	5	0.5	0	0.5	10	0	0
E.	peltatum	3	0	38	45	52	120	109	20	36	32
E.	brenesii	0	0	17	0	0	0	0	0	0	0
E.	bellermannianum	0	0	1	0	0	0	0.5	0	0	0
E.	siliquoides	0	0	0.5	0	0	0	0	2	1	14
P.	maculosa	0	0	46	5	0	0	0	0	0	0
P.	hirta	0	0	11	59	0	0	2	79	0	19
E.	gomezianum	0	0	42	57	13	0	2	162	0	0
E.	lingua	0	0	12	18	1	0	5	0	1	0
E.	moranii	0	0	0.5	4	10	8	1	0	4	7
E.	nigrescens	0	0	4	73	50	1	79	0.5	0.5	0.5
P.	peltilimba	0	0	16	271	224	0	339	75	89	26
P.	pittieri	0	0	0	2	1	14	0.5	0	0	0
E.	eximium	0	0	0	0.5	305	856	0.5	0	0	0
E.	auripilum	0	0	0	14	17	0.5	19	0	0	0
E.	standleyi	0	0	0	0.5	8	159	30	23	0	0
E.	ciliatum	0	0	0	10	6	0	48	3	111	171
P.	hernandiifolia	0	0	0	20	122	136	110	39	19	76
E.	gloeorrhizum	0	0	0	0	0.5	9	0	0	0	0

E.	decoratum	0	0	0	0	0.5	0	0	1	0	0
P.	palmana	0	0	0	0	102	1	50	111	0	0
E.	proximum	0	0	0	0	8	47	0.5	0.5	0	1
E.	phoras	0	0	0	0	0	5	0	0	0	0
P.	calvicaulis	0	0	0	0	0	0	60	67	107	111
E.	micropogon	0	0	0	0	0	0	0.5	1	2	7
E.	crinitum	0	0	0	0	0	0	0	14	0	38
E.	lalitae	0	0	0	0	0	0	0	12	0	22
P.	montium	0	0	0	0	0	0	0	2	14	1
E.	amygdalifolium	0	0	0	0	0	0	0	0	63	43
E.	heterochroum	0	0	0	0	0	0	0	0	5	42
E.	boragineum	0	0	0	0	0	0	0	0	0	1
E.	latum	0	0	0	0	0	0	0	0	0	11
E.	smithii	0	0	0	0	0	0	0	0	0	1

Table S8.
Species by site occurrence matrix for Baru. Each row is a species, each column a survey site, indicated by elevation in m.a.s.l. Numbers in each cell represent the average number of individuals of each species found each 30-minute interval of timed searches.

	species	3400	3000	2600	2000	1700	1500	1200	800
E.	furfuraceum	161.7	39.1	1.6	0	0	0	0	0
E.	gayanum	160.0	2.8	0	0	0	0	0	0
E.	conspersum	6.1	5.1	0.6	0	0	0	0	0
E.	“aff. brenesii”	5.6	0	0	0	0	0	0	0
E.	"blue tear"	0.4	5.5	0	0	0	0	0	0
E.	bellermannianum	0.2	0	0	2.2	0	0	0	0
E.	squamipes	0	8.2	0.5	0	0	0	0	0
E.	brenesii	0	3.8	31.2	0.6	0	0	0	0
E.	setigerum	0	0.1	5.1	0	0	0	0	0
P.	galioides	0	6	19.0	0	0	0	0	0
P.	quadrifolia	0	0	30.3	0	0	0	0	0
E.	subcuspidatum	0	0	0	7.3	2.5	0	0	0
E.	gomezianum	0	0	0	7.1	0.1	4.4	7.8	0
E.	corifolium	0	0	0	5.3	0.1	1.8	0	0
E.	auripilum	0	0	0	3.5	8.3	0.5	0	0
E.	paleaceum	0	0	0	0.2	0	0	0	0
E.	lloense	0	0	0	0.8	0	0	0	0
E.	glabellum	0	0	0	3.4	0	0.6	0	0
E.	lingua	0	0	0	2.1	0.1	3.2	1.1	0.1
E.	gloeorrhizum	0	0	0	2.1	4.3	0	0	0
E.	peltatum	0	0	0	2.4	4.4	0.8	0.8	2.3
E.	hammellianum	0	0	0	0.8	3.7	8.7	3.5	0.8
P.	palmana	0	0	0	12.5	0	0	0	0
P.	hernandifolia	0	0	0	0.1	11.1	6.9	0	1.8
E.	boquetense	0	0	0	0	17.5	19.3	6.1	0
E.	ciliatum	0	0	0	0	0.3	27.9	2	4
E.	proximum	0	0	0	0	0.2	0.3	0	0
E.	eximium	0	0	0	0	0.1	6.0	0	0
E.	doanense	0	0	0	0	5.3	5.5	27.2	5.9
E.	moranii	0	0	0	0	1.8	0.3	4.9	2.0
P.	calvicaulis	0	0	0	0	1.7	1.4	0	3.1
E.	caricifolium	0	0	0	0	0	13.9	13	0.1
E.	decoratum	0	0	0	0	0	0.3	0	0
P.	rotundifolia	0	0	0	0	0	0.2	0	0
E.	productum	0	0	0	0	0	0	0.4	2.8

E.	micropogon	0	0	0	0	0	0	5.8	0
E.	nigrescens	0	0	0	0	0	0	21.8	0.5
E.	phoras	0	0	0	0	0	0	0.3	0
P.	distachya	0	0	0	0	0	0	0.2	3.1
E.	heterochroum	0	0	0	0	0	0	0	2
E.	amygdalifolium	0	0	0	0	0	0	0	9.2
P.	urocarpoides	0	0	0	0	0	0	0	2.8
P.	serpens	0	0	0	0	0	0	0	3.3
E.	siliquoides	0	0	0	0	0	0	0	0.9
P.	flexinerva	0	0	0	0	0	0	0	0.8
E.	lalitae	0	0	0	0	0	0	0	0.5
E.	crinitum	0	0	0	0	0	0	0	0.3
E.	latum	0	0	0	0	0	0	0	0.3
