# Supplementary material for: Experimental evidence of climate change extinction risk in Neotropical montane epiphytes
Source: Nat Commun. 2024 Jul 18;15:6045. doi: 10.1038/s41467-024-49181-5 (PMC11258140; doi:10.1038/s41467-024-49181-5)
Supplement: Supplementary file 3 — Reporting Summary [file 41467_2024_49181_MOESM3_ESM.pdf]

Corresponding author(s): Emily Hollenbeck
Last updated by author(s): Feb 23, 2024

Reporting Summary

Nature Portfolio wishes to improve the reproducibility of the work that we publish. This form provides structure for consistency and transparency in reporting. For further information on Nature Portfolio policies, see our Editorial Policies and the Editorial Policy Checklist.

Statistics

For all statistical analyses, confirm that the following items are present in the figure legend, table legend, main text, or Methods section.

n/a	Confirmed	
☐	☒	The exact sample size (*n*) for each experimental group/condition, given as a discrete number and unit of measurement
☐	☒	A statement on whether measurements were taken from distinct samples or whether the same sample was measured repeatedly
☐	☒	The statistical test(s) used AND whether they are one- or two-sided<br>*Only common tests should be described solely by name; describe more complex techniques in the Methods section.*
☐	☒	A description of all covariates tested
☐	☒	A description of any assumptions or corrections, such as tests of normality and adjustment for multiple comparisons
☐	☒	A full description of the statistical parameters including central tendency (e.g. means) or other basic estimates (e.g. regression coefficient) AND variation (e.g. standard deviation) or associated estimates of uncertainty (e.g. confidence intervals)
☐	☒	For null hypothesis testing, the test statistic (e.g. F, t, r) with confidence intervals, effect sizes, degrees of freedom and P value noted<br>*Give P values as exact values whenever suitable.*
☒	☐	For Bayesian analysis, information on the choice of priors and Markov chain Monte Carlo settings
☒	☐	For hierarchical and complex designs, identification of the appropriate level for tests and full reporting of outcomes
☒	☐	Estimates of effect sizes (e.g. Cohen's *d*, Pearson's *r*), indicating how they were calculated

Our web collection on statistics for biologists contains articles on many of the points above.

Software and code

Policy information about availability of computer code

Data collection	Climate data were collected in the field using hardware and software from LogTags and Decagon.<br>LogTag hardware: HAXO8 data loggers;<br>Logtag software: "LogTag Analyzer for Windows", version downloaded in 2014.<br>Decagon hardware: VP-3 temperature/humidity sensor, LWS leaf wetness sensor, ECRN-100 precipitation gauge, EM50 data logger.<br>Decagon software: ECH2O Utility
Data analysis	Data analysis was performed using R: R Core Team (2022). R: A language and environment for statistical computing. R Foundation for Statistical Computing, Vienna, Austria. URL https://www.R-project.org/.<br>R package used in data analysis:<br>interval: Fay, M.P. & Shaw P.A. Exact and Asymptotic Weighted Logrank Tests for Interval Censored Data: The interval R package. J. Stat. Softw. 36, 1-34. (2010).<br><br>EstimateS software was used to create species-accumulation curves. Colwell, R.K. EstimateS: Statistical estimation of species richness and shared species from samples. Version 9. User's Guide and application published at: http://purl.oclc.org/estimates. (2013).

For manuscripts utilizing custom algorithms or software that are central to the research but not yet described in published literature, software must be made available to editors and reviewers. We strongly encourage code deposition in a community repository (e.g. GitHub). See the Nature Portfolio guidelines for submitting code & software for further information.

Data

Policy information about availability of data

All manuscripts must include a data availability statement. This statement should provide the following information, where applicable:

- Accession codes, unique identifiers, or web links for publicly available datasets
- A description of any restrictions on data availability
- For clinical datasets or third party data, please ensure that the statement adheres to our policy

Data and materials availability: All data for this study were collected in the field by the authors. The transplant data generated in this study have been deposited in an Open Science Framework repository and can be accessed at: https://osf.io/f54zy/?view_only=18f10d58704c4d9b8d3f13c0cc3cc6f1. The range survey data generated in this study are provided in the Supplementary Information (Tables S6-8). The processed source data for figures are provided with this paper.

Research involving human participants, their data, or biological material

Policy information about studies with human participants or human data. See also policy information about sex, gender (identity/presentation), and sexual orientation and race, ethnicity and racism.

Reporting on sex and gender	NA
Reporting on race, ethnicity, or other socially relevant groupings	NA
Population characteristics	NA
Recruitment	NA
Ethics oversight	NA

Note that full information on the approval of the study protocol must also be provided in the manuscript.

Field-specific reporting

Please select the one below that is the best fit for your research. If you are not sure, read the appropriate sections before making your selection.

☐ Life sciences ☐ Behavioural & social sciences ☒ Ecological, evolutionary & environmental sciences

For a reference copy of the document with all sections, see nature.com/documents/nr-reporting-summary-flat.pdf

Ecological, evolutionary & environmental sciences study design

All studies must disclose on these points even when the disclosure is negative.

Study description

A transplant experiment was performed using 15 species and 6 field sites. The treatment factors were the "transplant direction," which is defined as all the individual plants moving from one unique site to another unique site (e.g., Site 4 to Site 5). There were a total of 24 unique experimental transplant directions, including 6 transplant controls (i.e., cases where the origin and destination site were the same). Within each transplant direction, there were 4 sampling blocks, or experimental units. A sampling block was a wooden scaffold containing about 5 individuals of each target species, ranging from 1 to 6 species per origin site (see methods for details). The unit of replication was individual species moved to specific transplant and control sites; the number of replicates of each species at each transplant or control site varied between 5 and 22 individuals. Sampling blocks were placed at random distances along a 100m transect at each destination site. Transplants were collected from the understory and deposited in the understory.

For the survey portion of the study, standardized "timed walks" (each 30 minutes long) were used to count the presence and abundance (number of individuals counted) of all species in the target genera at all survey sites. These data were confirmed to be complete using Species-Accumulation Curves, and were used to estimate the elevational range of each unique species.

Research sample

The research sample was species in the genera Elaphoglossum and Peperomia. These genera were chosen because they have high species richness and turnover across the elevational gradients we studied, allowing us to include many species in our experiment, and also because they are epiphytes (plants that grow perched on the branches and trunks of other plants, without rooting in the ground) and thus can be transplanted more easily than terrestrial plants. 15 species were used in the transplant experiment, and 49 species were used for the final data analysis in the range surveys. The criteria for including species were: we used all species within these genera that were detected in the area, that were possible to identify to species level in the field, and (for the transplant experiment) that had enough natural abundance to collect 5-20 individuals per site without severely depleting the natural population. The sampled individuals included in the experiment are intended to represent the broader populations of these epiphytic species.

Sampling strategy

For the transplant experiment, species were selected that could be found growing naturally as understory epiphytes (on low

Sampling strategy	branches and trunks) in sufficient abundance to collect dozens of individuals from a small area; see methods for exact sample sizes. For the survey experiment, data were collected in 6-hour "timed walks" through the forest understory at each site, carried out in a zigzag pattern as to not cover the same area twice, while remaining within the target elevation. Timed walks were broken into 30-minute segments that were considered a single sample, so at least 12 samples were used per site. Species-Accumulation Curves were used to analyze these data to confirm that total diversity was captured. This sampling method was developed after many trials of different survey methods, including standardized surveys of tree branches (by climbing into the canopy) as well as understory plots of standard size. After several years of field work, we determined that timed walks detected the greatest number of species per site.
Data collection	Data were collected by ECH in the field. Quantitative and qualitative data were recorded by hand in field notebooks and then transferred to digital spreadsheets. Qualitative data were also collected in the form of photos of individual plants, sites, and scaffolds.
Timing and spatial scale	In the transplant experiment, plants were first transplanted onto scaffolds in July and August of 2014. After a 6-week acclimation period, scaffolds were moved to transplant sites in September 2014. The total spatial extent of individual sites ranged not more than 50m in elevation and not more than 400m in distance. The total spatial distance between transplant sites ranged from 800m to 4km. After transplant, the experimental plants were monitored (full data collection) once every 3 months for 3 years, ending in September 2017. At each site, 4 replicate scaffolds were placed along a 100m transect at positions determined by a random number generator.
Data exclusions	Some data were excluded from the transplant experiment when the sample size for a particular species was so low per replicate that statistical analysis was not possible. Some species were excluded from the final survey data if the taxonomic identification to species level was at all uncertain, e.g. because there are disagreements in the taxonomic literature, or it was difficult to distinguish between closely related species in the field.
Reproducibility	The overall transplant experiment was only performed once, because it was very time- and labor-intensive: it took 3 years and required the collection of over 1000 plants. There was replication within the experiment. Four replicate microsites at each transplant site were used to determine statistical significance.
Randomization	Individual plants were randomly placed onto transplant scaffolds.<br>Replicate transplant scaffolds were placed at microsites that were randomly distributed along 100m transects at destination sites, with positions determined by a random number generator.
Blinding	Blinding was not possible as the researcher had to reliably identify all individuals to species level in the field, and had to return to the same plants (in the transplant experiment) for repeated data collection.

Did the study involve field work? ☒ Yes ☐ No

Field work, collection and transport

Field conditions	Field sites ranged from sea level to 3400m.a.s.l. in the tropical mountains of Costa Rica and Panama. Sites included tropical rain forest, premontane rain forest, premontane moist forest, montane rain forest, and paramo. In conventional terms, these were rainforest and cloud forest sites with high rainfall and moisture, relatively constant temperatures throughout the year, and in some cases, significant cloud immersion.
Location	Costa Rica: Monteverde and Volcan Barva. Panama: Volcan Baru and La Fortuna. All sites between 9-10 degrees latitude and between 0-3400m.a.s.l.
Access & import/export	All field work was carried out with permission from land owners (private reserves, national parks, and private individuals; see supplementary text for lists), and with research permits for Emily Hollenbeck from Costa Rica's Sistema Nacional de Areas de Conservación from 2013-17 (Scientific Passport no. 04964) and Panama's Ministerio de Ambiente in 2016. Collections of some individual plants were performed in order to confirm taxonomic identification, but no specimens were exported. Collections were deposited in the National Herbarium of Costa Rica, the herbarium of the University of Panama, and the herbarium of the Smithsonian Tropical Research Institute in Panama.
Disturbance	Some disturbance was caused by collection of individual plants, as well as by the researchers moving through the forest understory. We always attempted to minimize any extraneous disturbance of vegetation during movement through the understory. Collections of plants always removed the minimum number of plants possible to achieve the experimental goals (e.g. 2 per species for herbarium specimens to confirm identification; 20 per species per site for transplant experiment)

Reporting for specific materials, systems and methods

We require information from authors about some types of materials, experimental systems and methods used in many studies. Here, indicate whether each material, system or method listed is relevant to your study. If you are not sure if a list item applies to your research, read the appropriate section before selecting a response.

Materials & experimental systems

n/a	Involved in the study
☒	☐ Antibodies
☒	☐ Eukaryotic cell lines
☒	☐ Palaeontology and archaeology
☒	☐ Animals and other organisms
☒	☐ Clinical data
☒	☐ Dual use research of concern
☐	☒ Plants

Methods

n/a	Involved in the study
☒	☐ ChIP-seq
☒	☐ Flow cytometry
☒	☐ MRI-based neuroimaging

Dual use research of concern

Policy information about dual use research of concern

Hazards

Could the accidental, deliberate or reckless misuse of agents or technologies generated in the work, or the application of information presented in the manuscript, pose a threat to:

No	Yes
☒	☐ Public health
☒	☐ National security
☒	☐ Crops and/or livestock
☒	☐ Ecosystems
☒	☐ Any other significant area

Experiments of concern

Does the work involve any of these experiments of concern:

No	Yes
☒	☐ Demonstrate how to render a vaccine ineffective
☒	☐ Confer resistance to therapeutically useful antibiotics or antiviral agents
☒	☐ Enhance the virulence of a pathogen or render a nonpathogen virulent
☒	☐ Increase transmissibility of a pathogen
☒	☐ Alter the host range of a pathogen
☒	☐ Enable evasion of diagnostic/detection modalities
☒	☐ Enable the weaponization of a biological agent or toxin
☐	☐ Any other potentially harmful combination of experiments and agents

Plants

Seed stocks	See statement below
Novel plant genotypes	See statement below
Authentication	Plant specimens were collected from the field. Live specimens were collected and transplanted in Monteverde, Costa Rica. Collections and transplantations were performed in July-September 2014. Epiphytic plants were collected growing in the understory, were transplanted onto wooden scaffolds, and moved to destination sites as described above and in the methods section. Transplant sites were all within 4km and 600m a.s.l. of each other.<br>Plant specimens were collected and dried for herbarium records (dead specimens) in several locations in Costa Rica (Monteverde, and Volcan Barva in Braulio Carrillo National Park, ranging from 0-2800m.a.s.l.), as well as Panama (La Fortuna of the Smithsonian Tropical Research Institute, and Volcan Baru National Park). Specimens were deposited in herbaria and under research permits as described above. No authentication, seeds, or genotypes were involved.
